# Acute Abdomen Due to Spontaneous Splenic Rupture in an Adult Polycystic Kidney Disease Patient: A Case Report

**DOI:** 10.1155/cris/9328289

**Published:** 2026-11-09

**Authors:** Suhaila Ahamed Ahmed, Maryam Khan, Fathima Shabrina Rizni, Labib Sallam Darwish Al Ozaibi, Rafay Gul

**Affiliations:** ^1^ General Surgery Rashid Hospital Dubai UAE dha.gov.ae; ^2^ Interventional Radiology Rashid Hospital Dubai UAE dha.gov.ae

## Abstract

Adult polycystic kidney disease (APKD) is a hereditary disorder characterized by renal and extrarenal manifestations. While renal complications are well recognized, spontaneous splenic rupture in APKD is exceptionally rare, with the available literature largely limited to isolated case reports; however, atraumatic splenic rupture overall has been estimated to account for only 0.1–0.5% of splenic ruptures and carries a reported mortality rate of ~12.2%, largely due to delayed diagnosis and uncontrolled intraperitoneal hemorrhage. We report the case of a 68‐year‐old male with APKD who presented with severe left upper quadrant (LUQ) abdominal pain, fever, and jaundice. Initial evaluation demonstrated splenomegaly with splenic lacerations, as well as a distal common bile duct (CBD) stone causing obstructive jaundice. Despite antibiotic therapy, the patient developed persistent fever and an acute drop in hemoglobin, raising concern for ongoing intra‐abdominal hemorrhage. Endoscopic retrograde cholangiopancreatography (ERCP) was performed for CBD stone extraction and biliary stent placement. Due to continued bleeding, splenic artery embolization (SAE) was subsequently performed, achieving hemostasis while preserving splenic function. This case highlights the importance of maintaining clinical vigilance for rare but potentially fatal extrarenal complications in patients with APKD. In patients with this condition who present with abdominal pain, fever, jaundice, or unexplained anemia, splenic pathology should be considered in the differential diagnosis. Early diagnosis and timely multidisciplinary intervention, including endoscopic and interventional radiology techniques, can be life‐saving and may avoid the need for splenectomy.

## 1. Introduction

Adult polycystic kidney disease (APKD) is a hereditary disorder characterized by progressive cyst formation, predominantly affecting the kidneys and liver, with a broad spectrum of extrarenal manifestations [1, 2]. Although renal complications are the most frequently recognized, clinically significant abdominal complications may also arise from splenic and hepatobiliary involvement, creating complex diagnostic and therapeutic challenges.

Splenic involvement in APKD is rare but may increase the risk of spontaneous rupture, resulting in potentially fatal hemorrhage or peritonitis [3, 4]. In hemodynamically stable patients, nonoperative management is generally preferred (success rates 60%–95%), with splenic artery embolization (SAE) serving as an organ‐preserving alternative to surgery in selected cases, particularly when there is evidence of ongoing bleeding or vascular injury [5–7].

Hepatobiliary complications are also recognized in APKD and may present with obstructive jaundice, most commonly due to hepatic cyst‐related biliary compression, although other causes such as choledocholithiasis should also be considered [8–10]. However, the coexistence of spontaneous splenic rupture and obstructive jaundice with cholangitis in a patient with APKD is rarely described, and optimal multidisciplinary management in this setting remains insufficiently characterized.

We present a rare case of APKD complicated by spontaneous splenic rupture and obstructive jaundice secondary to choledocholithiasis with cholangitis, successfully managed with endoscopic intervention and SAE.

## 2. Case Presentation

A 68‐year‐old male with a history of APKD, polycystic liver disease, and type 2 diabetes mellitus, and a history of chronic smoking presented with severe left upper quadrant (LUQ) pain for 2 days, associated with fever, chills, anorexia, scleral icterus, clay‐colored stools, and dark urine. He denied any trauma, recent travel, or significant weight loss.

The patient’s medical history included a previous hepatic abscess requiring percutaneous drainage. His family history was remarkable for APKD in his daughter, though no genetic testing had been conducted. There was no known history of pre‐existing splenic cysts, splenic lesions, or vascular abnormalities.

On examination, the patient was alert and hemodynamically stable, with a Glasgow Coma Scale of 15. LUQ tenderness was noted without guarding or rebound tenderness, and the Murphy’s sign was negative.

Laboratory investigations revealed leucocytosis with a white blood cell count of 14.0 × 10^3^/uL, low hemoglobin (11.8 g/dL), and low hematocrit (34.9%). Inflammatory markers were markedly elevated, with C‐reactive protein of 328.9 mg/L and procalcitonin of 2.67 ng/mL. Liver function tests demonstrated an obstructive pattern, with alkaline phosphatase of 143 U/L, total bilirubin of 7.23 mg/dL, and direct bilirubin of 5.36 mg/dL.

Contrast‐enhanced computed tomography (CT) of the abdomen was performed, as shown in Figures 1–3. CT demonstrated splenomegaly with multiple splenic lacerations and contusions, associated with perisplenic and intra‐abdominal hematoma, consistent with a high‐grade splenic injury, likely American Association for the Surgery of Trauma (AAST) grade III–IV. No history of trauma was reported, supporting the diagnosis of spontaneous splenic injury. Hepatobiliary findings included acute cholecystitis with possible intraluminal calculi or polyps, a distal common bile duct (CBD) calculus causing mild biliary obstruction, and mild intrahepatic biliary dilatation. Multiple calcifications were also noted along the walls of hepatic cysts, in keeping with the underlying polycystic liver disease. Renal findings included bilateral multiple renal cysts and calculi, consistent with the patient’s known APKD. Mild free fluid was present in the pelvis. Overall, the clinical, laboratory, and CT findings supported a diagnosis of sepsis secondary to acute cholangitis and choledocholithiasis, Tokyo Classification grade II, with concurrent spontaneous high‐grade splenic injury.

**Figure 1:**
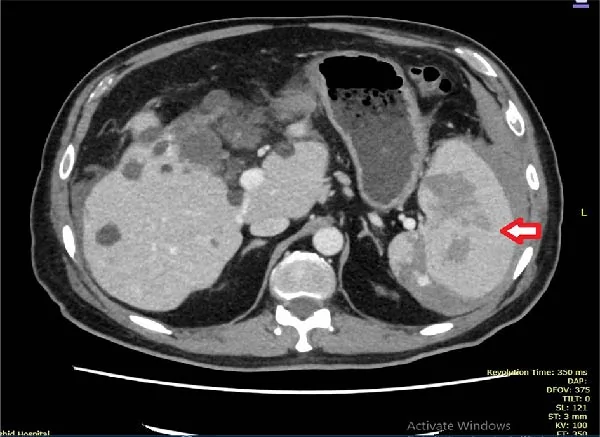
CT abdomen with contrast (axial view). Splenomegaly with multiple lacerations and contusions with peri splenic and intra‐abdominal hematoma as shown by the red arrow.

**Figure 2:**
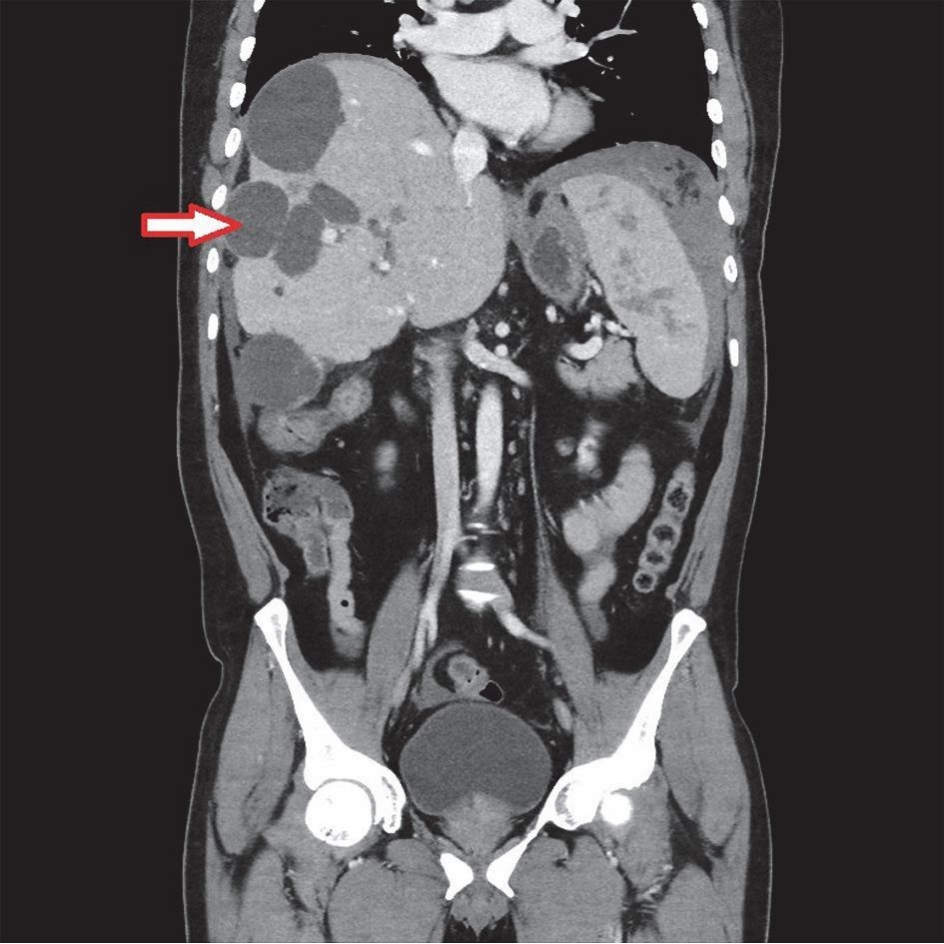
CT abdomen with contrast (coronal view). Distal CBD calculus accompanied by mild intrahepatic biliary dilatation and multiple calcifications in the cystic walls of the liver marked by the red arrow.

**Figure 3:**
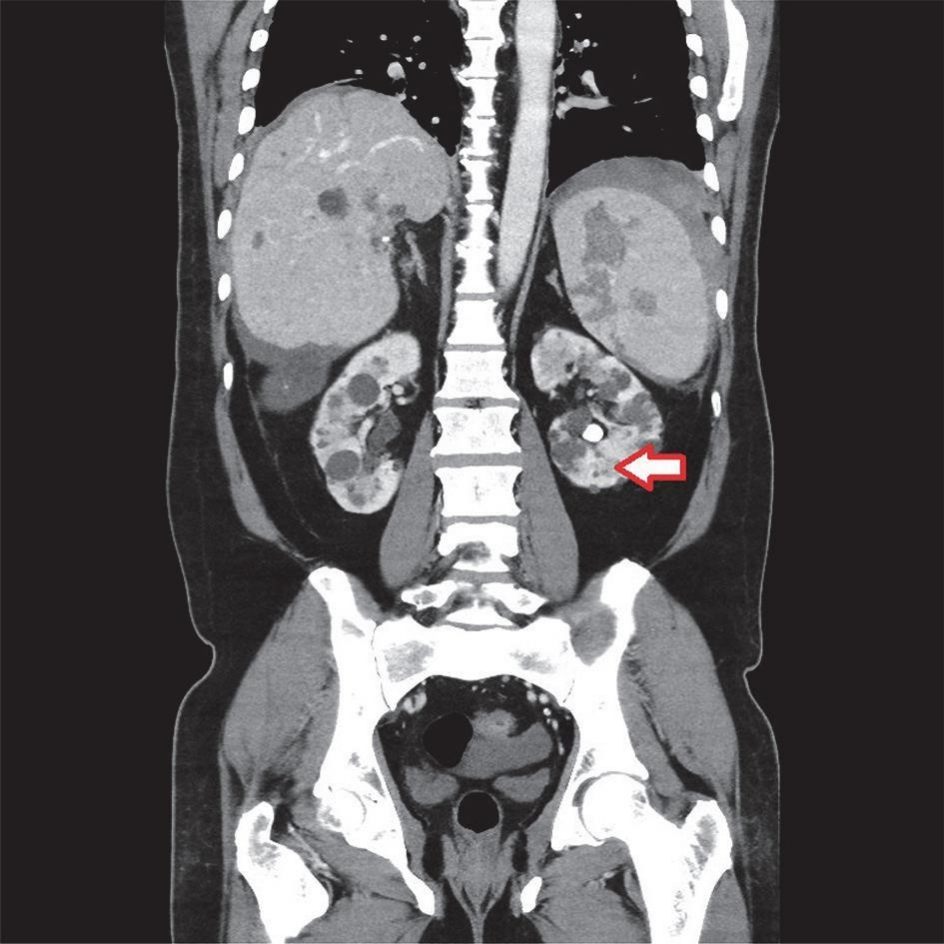
CT abdomen with contrast (coronal view). Multiple bilateral renal cysts are marked by the red arrow.

On the day of admission, empiric broad‐spectrum antibiotics were initiated, and endoscopic retrograde cholangiopancreatography (ERCP) with sphincterotomy, CBD stone extraction, and biliary stent placement was performed. The high‐grade splenic injury was initially managed nonoperatively because the patient was hemodynamically stable, had no peritonitic signs, and had no immediate evidence of uncontrolled active arterial hemorrhage, making close monitoring with serial clinical assessment and hemoglobin measurements appropriate despite the presence of splenomegaly.

On the third day of admission, the patient developed a sudden drop in hemoglobin, prompting urgent repeat imaging. Repeat imaging demonstrated an abnormally dilated peripheral intrasplenic vessel with minimal contrast pooling on the delayed phase, suggestive of slow‐flow venous oozing rather than brisk arterial extravasation. Selective splenic angiography was subsequently performed and demonstrated abnormal vascularity with suspected contrast extravasation arising from segmental splenic artery branches, as shown in Figure 4, corresponding to the suspected bleeding site. Super‐selective catheterization of the feeding arterial branch was achieved, followed by embolization. Postembolization angiography confirmed complete occlusion of the feeding arteries with no further contrast pooling, indicating a technical success. Following embolization, the patient’s hemoglobin stabilized.

**Figure 4:**
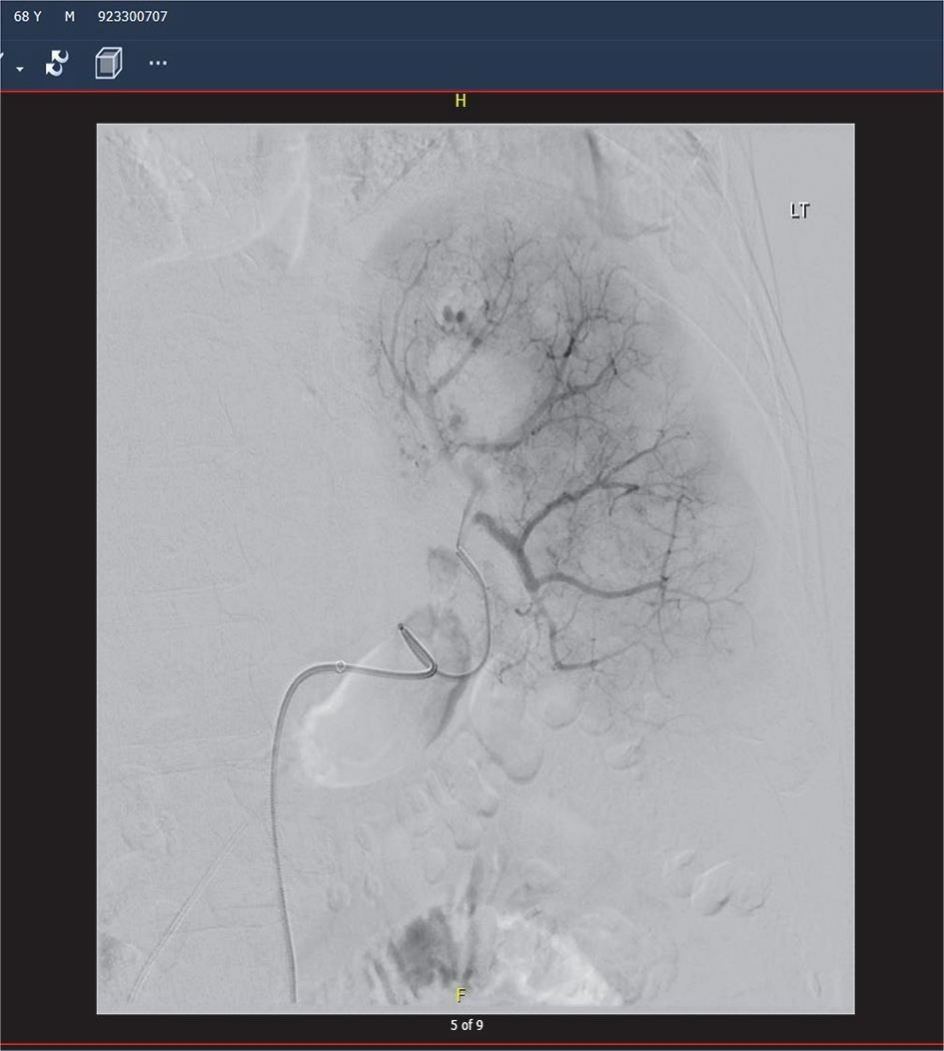
Selective splenic angiogram. Abnormal vascularity and potential extravasation from segmental splenic artery branches.

Following an extended hospital stay and multidisciplinary management, including targeted antibiotics, ERCP, and SAE, the patient was discharged in stable condition. At discharge, he was clinically well, afebrile, with improving bilirubin and inflammatory markers, and stable hemoglobin.

## 3. Discussion

APKD is a systemic hereditary disorder characterized by progressive cyst formation in the kidneys and other organs, particularly the liver. Although hepatic and renal complications are well recognized, splenic manifestations are uncommon and remain poorly characterized in the literature. This case adds to the existing literature by documenting an unusual dual presentation of spontaneous high‐grade splenic injury and obstructive jaundice with cholangitis in an elderly patient with APKD, creating competing priorities of infection control, biliary decompression, hemorrhage surveillance, and splenic preservation, and demonstrating successful multidisciplinary management using a spleen‐preserving approach.

Splenic involvement in APKD is rarely reported and remains poorly characterized compared with renal, hepatic, intracranial, and cardiovascular manifestations. Reported splenic abnormalities include splenic cysts, splenomegaly, and rare vascular lesions such as splenic artery aneurysms, suggesting that splenic pathology may represent an underrecognized component of the systemic cystic and vascular phenotype of APKD [11]. However, most available data are derived from isolated case reports or imaging‐based observations rather than large prospective cohorts, and the true prevalence, natural history, and rupture risk of splenic lesions in APKD remain undefined. Although spontaneous splenic rupture in APKD is not well quantified, rupture of a pathologically vulnerable spleen may result in acute hemodynamic compromise, hemoperitoneum, and fatal hemorrhage if not promptly recognized, as demonstrated in recent reports of spontaneous splenic rupture in other systemic diseases with underlying splenic parenchymal vulnerability [12–14]. In our patient, the absence of trauma, the presence of splenomegaly, multiple splenic lacerations, perisplenic and intra‐abdominal hematoma, and subsequent hemoglobin drop supported the diagnosis of spontaneous splenic injury. Importantly, there was no known history of pre‐existing splenic cysts, splenic lesions, or vascular abnormalities, making this presentation particularly unusual.

Initial nonoperative management was appropriate because the patient was hemodynamically stable, had no peritonitic signs, and showed no immediate evidence of uncontrolled arterial bleeding. In such patients, nonoperative management is generally preferred, whereas surgical intervention, including splenorrhaphy or splenectomy, is usually reserved for hemodynamic instability, failed conservative management, or extensive transfusion requirements. However, the sudden hemoglobin drop on day three prompted repeat imaging, which demonstrated a peripheral intrasplenic vascular abnormality with delayed contrast pooling; selective angiography confirmed abnormal vascularity with suspected extravasation from segmental splenic artery branches. Given the role of SAE as an adjunct to nonoperative management, with reported spleen preservation rates of 87.1% compared with 32.1% for operative management and primary success rates of 80.9%, increasing to 88.1% after repeat intervention when required [7, 8], super‐selective embolization was performed. This achieved hemostasis while preserving splenic tissue, avoiding splenectomy and its associated long‐term infectious risks, and supporting the value of early repeat imaging and interventional radiology in stable patients with suspected ongoing hemorrhage.

The concomitant obstructive jaundice and cholangitis further distinguishes this case. Hepatobiliary disease is a recognized extrarenal manifestation of APKD, usually related to polycystic liver disease and anatomical distortion. In this patient, CT demonstrated a distal CBD stone with mild biliary obstruction and intrahepatic biliary dilatation, supporting the diagnosis of choledocholithiasis complicated by cholangitis. ERCP with sphincterotomy, stone extraction, and biliary stenting provided timely source control. The coexistence of cholangitis and splenic hemorrhage created a high‐risk scenario in which delayed recognition of either pathology could have led to clinical deterioration.

The patient’s chronic smoking history was recorded as part of his baseline risk profile; however, there was no evidence that smoking directly contributed to the acute splenic rupture or biliary obstruction in this case. Therefore, it was not considered a major explanatory factor in the pathogenesis of the presenting illness.

This case highlights several practical lessons. First, abdominal pain in APKD should not be attributed solely to renal or hepatic cyst complications, particularly when pain is localized to the LUQ or accompanied by anemia. Second, obstructive jaundice in APKD requires careful evaluation for both cyst‐related compression and common biliary causes such as choledocholithiasis. Third, early CT imaging, close hemoglobin monitoring, and timely repeat imaging can identify evolving hemorrhage before hemodynamic collapse occurs. Finally, selective SAE can be a decisive, organ‐preserving intervention in selected stable patients with ongoing splenic bleeding. Overall, this case reinforces the importance of multidisciplinary management involving gastroenterology, surgery, interventional radiology, nephrology, and hepatology in complex APKD presentations.

## Funding

No funding was received for this study.

## Consent

Informed consent was obtained from the patient.

## Conflicts of Interest

The authors declare no conflicts of interest.

## Data Availability

The data that support the findings of this study are available from the corresponding author upon reasonable request.
